# Do Postoperative Orthopedic Patients Accurately Report Their Narcotic Medication Usage Using the Interventional Pain Assessment Drugs Scale?

**DOI:** 10.7759/cureus.85852

**Published:** 2025-06-12

**Authors:** Tyler Forbes, Joseph Cavataio, Jason Salvato, Lauryn Boggs, Alqasim Elnaggar, Rahul Vaidya

**Affiliations:** 1 Department of Orthopedic Surgery, Wayne State University Detroit Medical Center, Detroit, USA

**Keywords:** opioid medication, orthopaedic and trauma, patient-centred care, patient engagment, post-operative opioids

## Abstract

Introduction: Accurately assessing opioid consumption is critical for effective postoperative pain management and reducing misuse. While prescription monitoring tools like the Michigan Automated Prescription System (MAPS) provide objective data, they may not reflect actual patient behavior. This study evaluates the accuracy of self-reported opioid use using the Interventional Pain Assessment-Drugs (IPA-D) scale.

Methods: A prospective IRB-approved study enrolled 753 postoperative orthopedic patients at a Level I trauma center from June 2022 to June 2023. Participants completed the IPA-D survey to self-report opioid usage, which was stratified into five classes based on morphine milligram equivalents (MMEs). These reports were compared to MAPS prescription data to determine classification accuracy.

Results: The IPA-D scale demonstrated high accuracy. Classes A and B showed 100% accuracy with 0.0 MMEs. Class C (1-30 MMEs) had an accuracy of 99.3% with an average MME of 17.3. Class D (31-79 MMEs) reported 96.6% accuracy with an average MME of 42.81. Class E (≥80 MMEs) showed 94.4% accuracy and an average MME of 140.83.

Conclusion: The IPA-D scale enables reliable patient self-reporting of opioid use across most MME categories. It offers a practical, accurate tool for real-time opioid monitoring in clinical settings and may serve as a valuable complement to prescription databases like MAPS.

## Introduction

The opioid epidemic remains one of the most pressing public health crises in the United States, with millions of individuals affected by opioid misuse, addiction, and overdose [1-3]. As healthcare providers navigate the delicate balance between effective pain management and minimizing opioid misuse, there is a growing need for reliable tools to track opioid consumption [4,5]. The Centers for Disease Control and Prevention (CDC) recognized the need for a national guideline on pain management that could improve appropriate opioid prescribing while minimizing opioid-related risks and released the concept of morphine milligram equivalents (MMEs), a standardized metric that helps assess the potency and dosage of opioids a patient is taking [6]. Accurate MME reporting is essential for safely managing opioid prescriptions and preventing the risks of overuse and addiction [7,8].​ 

Currently, tools such as the Michigan Automated Prescription System (MAPS) are widely used by healthcare providers to track controlled substance prescriptions, including opioids [9].​​ MAPS monitors and provides precise MME data based on the narcotics patients are prescribed to prevent drug abuse and diversion at the prescriber, pharmacy, and patient levels [9]. While prescription monitoring programs like MAPS offer accurate information regarding prescribed doses, they do not account for real-time variations in patient consumption, such as patients under-reporting or not adhering to prescribed regimens [6]. This limitation highlights the need for complementary methods to assess opioid use based on patient-reported data. 

The Interventional Pain Assessment-Drugs (IPA-D) scale presents a potential solution for bridging the gap between prescribed and actual opioid use. The IPA-D scale allows healthcare providers to assess patients’ opioid consumption by interpreting self-reported usage patterns [10]. Unlike MAPS, which provides exact MME values, the IPA-D scale categorizes opioid use into MME ranges and demonstrates the ability to evaluate pain perception and medication usage and monitor pain management efficacy [11]. Previous literature has emphasized a patient preference for the IPA-D scale in comparison to the Numerical Rating Scale (NRS), the pain intensity scale of 0 (no pain) to 10 (worst pain), attributed to its simplicity, sensitivity to changes in patient’s pain experience, and its guidance during treatment [10-12]. This approach offers a practical way to estimate opioid usage when precise prescription data may not reflect real-time patient consumption behavior.

Despite the potential utility of patient-reported outcome measures like the IPA-D scale, concerns remain regarding the accuracy of self-reported medication usage. Studies have demonstrated that many patients struggle to accurately recall or update their medication lists, leading to discrepancies in dosage at admission that can contribute to issues of underdosing or overdosing and significant clinical consequences [13-18]. However, various studies also highlight patient self-reported data to be more reliable when compared to electronic health records (EHRs), contributing to the overall heterogeneity of patients’ accuracy in reporting, updating, and contributing to their medication usage [19-21]. These findings underscore the importance of validating patient-reported opioid use to ensure it aligns with objective prescription data.

This study evaluates the accuracy of patients’ self-reported daily MME usage using the IPA-D scale by comparing it to objective data from MAPS across three orthopedic clinics at a level 1 trauma center specializing in spine, trauma, and hip and joint replacement. Recognizing the potential discrepancies between self-reported and actual prescription data, the study aims to assess how accurately patients can reflect their true daily MME usage. We hypothesize that patients will demonstrate a high degree of accuracy in reporting their daily MMEs through the IPA-D scale.

## Materials and methods

An IRB-approved prospective study was conducted in the Department of Orthopedics at the Detroit Medical Center, a level 1 trauma center, in Detroit, Michigan, USA. The study included postoperative orthopedic patients aged 18 years or older who provided informed consent. Patients who declined to participate or were under the age of 18 were excluded.

A total of 753 patients were enrolled between June 2022 and June 2023 during routine follow-up visits at an orthopedic clinic after operative treatment for orthopedic trauma. A trained research assistant obtained verbal consent from each patient and administered the Instructional Pain Assessment-Drugs (IPA-D) survey. The survey collected demographic information, including gender, age, type of surgery (classified using ICD-10 and CPT codes), time from surgery, and general health status.

As shown in Table 1, the patient-reported opioid usage was stratified into five classes based on the type and frequency of medication use. Class A included patients who reported taking no medication (0 MMEs), while Class B included those who used over-the-counter medications only, also corresponding to 0 MMEs. Class C consisted of patients who occasionally used short-acting narcotics (1-30 MMEs). Class D included those who regularly used short-acting narcotics (31-79 MMEs), and Class E represented patients who used long-acting narcotics in combination with short-acting medications for breakthrough pain, with usage of 80 or more MMEs.

**Table 1:** Medication classification A detailed description of each medication class providing the range of morphine milligram equivalents (MMEs) they are meant to estimate and the types of medications that would fall into each category.

Class	Daily Morphine Milligram Equivalents (MMEs)	Types of Medication
A: No Medication	0	N/A
B: Over-the-counter	0	N/A
C: Occasional use of short-acting narcotics	1-30	Schedule II and IV drugs (tramadol, codeine, hydrocodone, or oxycodone)
D: Regular use of short-acting narcotics	31-79	Schedule II drugs (hydrocodone, or oxycodone)
E: Long-acting narcotics with breakthrough short-acting	80+	Schedule II drugs (hydromorphone, methadone, morphine, hydrocodone, or oxycodone hydrochloride)

Actual opioid prescription data, including daily morphine milligram equivalents (MMEs), were obtained from the Michigan Automated Prescription System (MAPS). Patient-reported data from the IPA-D survey were compared to MAPS data to assess reporting accuracy. The two primary outcomes analyzed were the accuracy of IPA-D classifications (percentage of correct matches) and the average MMEs within each class.

Statistical analysis

Descriptive statistics were used to summarize patient demographics, body mass index (BMI), and follow-up duration. Frequencies and percentages were reported for categorical variables, including IPA-D classification and gender.

To evaluate the accuracy of self-reported opioid use, each patient’s IPA-D classification was compared with their MAPS-derived average daily MME. Accuracy was defined as the proportion of patients whose reported class aligned with their actual MME category. For classes C through E, mean MME values and 95% confidence intervals were calculated to assess the consistency of patient self-reporting.

All statistical analyses were performed using IBM Corp. Released 2022. IBM SPSS Statistics for Windows, Version 28. Armonk, NY: IBM Corp.

## Results

The 753 patients included in this study consisted of patients who have experienced postoperative measures and care. This study consisted of 441 (58.5%) female and 312 (41.5%) male patients, with an average body mass index (BMI) of 31.72 kg/m², and was a racially/ethnically representative population for the Detroit Medical Center’s surrounding population. The post-surgical follow-up times varied widely, ranging from one-day post-op to 10 years after surgery, with a median follow-up time of 78 days post-surgery. The number of patients falling into each IPA-D medication class, respectively from A to E, are as follows: 154 (20.4%), 242 (32.1%), 281 (37.3%), 59 (7.8%), and 18 (2.4%). Patient demographics are summarized in Table 2.

**Table 2:** Patient demographics and opioid use by IPA-D class Summary of sex, body mass index (BMI), and opioid use classification in 753 postoperative orthopedic trauma patients. Values are presented as N (%).

Demographic	Value
Total Patients	753
Sex	
– Female	441 (58.5%)
– Male	312 (41.5%)
Average BMI (kg/m²)	31.72
IPA-D Medication Class Distribution	
– Class A (No medication; 0 MMEs)	154 (20.4%)
– Class B (Over-the-counter; 0 MMEs)	242 (32.1%)
– Class C (Occasional short-acting; 1–30 MMEs)	281 (37.3%)
– Class D (Regular short-acting; 31–79 MMEs)	59 (7.8%)
– Class E (Long-acting with breakthrough; ≥80 MMEs)	18 (2.4%)

The IPA-D scale demonstrated a high overall accuracy, as shown in Table 3. Particularly, classes A and B each had a reporting accuracy of 100% across each specialty, with MME scores of 0.0 for all patients. Class C patients had MME scores of 17.3 (95% CI ± 1.1) with an accuracy of 99.3%. Class D patients had an average MME score of 42.81 (95% CI ± 1.85) with an accuracy of 96.6%. Class E patients had an average MME score of 140.83 (95% CI ± 25.91) with an accuracy of 94.4%.

**Table 3:** Patient's self-reported accuracy Accuracy of each patient’s placement into their respective medication class based on their self-reported medication use.

Class	n Total	n Incorrect	Percent Correct (%)	Mean MME (95% CI)
A	153	0	100%	0 (± 0)
B	241	0	100%	0 (± 0)
C	280	2	99.30%	17.3 (± 1.1)
D	58	2	96.60%	42.81 (± 1.85)
E	18	1	94.40%	140.83 (± 25.91)

The two patients incorrectly categorized into Class C have MME scores of 45 and 67.5. The two patients incorrectly categorized into Class D have MME scores of 15.0 and 30.0. The one incorrectly categorized patient in Class E has an MME score of 80 and was previously on methadone prior to surgery.

## Discussion

This study evaluated the accuracy of patients reporting their daily opioid usage by utilizing the IPA-D scale in estimating daily MMEs compared to the MAPS and provided insights into the utility of the IPA-D scale as a complementary tool for opioid monitoring. The IPA-D scale demonstrated that patients who needed surgical care exhibited excellent accuracy (100%) in reporting classes A (no opioid use) and B (over-the-counter medications) in the datasets. This consistency highlights the scale's effectiveness in identifying patients with minimal or no opioid exposure, making it a valuable tool for assessing low-risk populations. Similarly, class C (occasional short-acting narcotics) showed impressive accuracy, with patients reporting an average of 99.3% accuracy. Class D (regular short-acting narcotics) also demonstrated high accuracy, with an average score of 96.6%, reflecting strong self-reporting of opioid usage. Both narcotic groups C and D exhibited high accuracy, with only minor discrepancies. In contrast, class E (long-acting narcotics with breakthrough short-acting) had a slightly lower accuracy of 94.4%, showing the most variability among all narcotic classes. These findings suggest that patients can accurately and effectively self-report their opioid use using the IPA-D scale, particularly for routine pain management and medication consumption patterns.

The most significant variability in the entire dataset was observed in class E (long-acting narcotics with breakthrough short-acting), which corresponds to patients with the highest levels of opioid consumption. These discrepancies likely reflect the complexities of accurately capturing opioid use in high-dose patients, who may have greater variability in adherence, reporting, and treatment regimens, which can be influenced by factors such as the severity of pain, psychological comorbidities, and the presence of substance use disorders [22,23]. High-dose users also represent a population at greater risk for misuse and overdose, underscoring the need for improved monitoring strategies in this subgroup [24]. These findings also point to the potential for under-reporting or misclassification in class E due to patient recall bias, fear of stigma, or misunderstanding of prescribed dosages [25,26].

Several studies have explored the accuracy of patient-reported opioid usage, with a focus on enhancing opioid monitoring in clinical practice. These studies examine the role of self-reporting opioid usage as it relates to pain management, adjusting treatment perceptions, and identifying misuse or overdose [21,27-29]. These studies found that patients are a reliable source of reporting drug usage, up until a certain point of drug category or usage, or other factors cause misreporting, such as depression [21,29]. While these previous studies relied on patients' self-reports to assess the accuracy of their disclosed drug usage, biological verification methods such as blood toxicology or urine drug screening are often impractical in routine clinical settings. Our findings suggest that when patients are provided with a structured self-reporting tool like the IPA-D scale, it can serve as a reliable alternative and can be particularly effective when combined with clinician oversight and supplementary risk assessment measures.

Despite its strengths, this study has several limitations that should be acknowledged. The first limitation is that patients in Michigan are aware of the MAPS system, which monitors prescription dosages; this awareness may encourage patients to be more truthful about their opioid use. Lastly, the study did not explore the impact of demographic variables, such as age, gender, or socioeconomic status, which may influence the accuracy of patient-reported data.

Future research should prioritize prospective validation of the IPA-D scale in diverse patient populations, including non-English-speaking individuals and those with varying levels of health literacy. Another future study that can be performed is to compare how patients report their pain as it relates to their drug usage. Additionally, for populations at the extremes of opioid use, refining and expanding the MME categories within class E could better capture the wide dosage ranges observed in this group. This refinement may help identify external factors, such as variations in prescribing practices, patient demographics, or institutional protocols, that may influence the scale's accuracy.

## Conclusions

In conclusion, this study demonstrates the reliability of the IPA-D scale in enabling patients to accurately report their daily opioid usage. The findings suggest that physicians can rely on patient self-reports, especially for individuals not using opioids, using over-the-counter medications, or taking occasional or regular short-acting opioids. However, the accuracy of self-reports becomes less certain for patients on long-acting narcotics or breakthrough short-acting opioids. The IPA-D scale proves to be an efficient tool for real-time assessment of opioid consumption, effectively capturing patients' lived experiences. This approach is particularly valuable in outpatient settings, where access to prescription monitoring databases may be limited. Overall, the IPA-D scale effectively bridges the gap between prescribed and actual opioid use, offering significant potential for improving opioid management.
